# First description of an infection by *Acinetobacter pitti / lactucae* subcomplex in Peru

**DOI:** 10.17843/rpmesp.2023.403.12721

**Published:** 2023-09-25

**Authors:** Carla Andrea Alonso, Jorge Choque-Matos, Fernando Guibert, Beatriz Rojo-Bezares, María López, Rocio Egoávil-Espejo, Patricia Gonzales, Carmen Valera-Krumdieck, María J. Pons, Yolanda Saénz, Joaquim Ruiz

**Affiliations:** 1 Department of Biomedical Diagnostics, Microbiology Laboratory, San Pedro Hospital, Logroño, Spain. Department of Biomedical Diagnostics Microbiology Laboratory San Pedro Hospital Logroño Spain; 2 Regenerative Medicine Group, Universidad Científica del Sur, Lima, Peru. Universidad Científica del Sur Regenerative Medicine Group Universidad Científica del Sur Lima Peru; 3 Research Group on Dynamics and Epidemiology of Antimicrobial Resistance - “One Health”, Universidad Científica del Sur, Lima, Peru. Universidad Científica del Sur Research Group on Dynamics and Epidemiology of Antimicrobial Resistance - “One Health” Universidad Científica del Sur Lima Peru; 4 Molecular Microbiology Area, Biomedical Research Center of La Rioja, Spain, Logroño, Spain. Molecular Microbiology Area Biomedical Research Center of La Rioja Logroño Spain; 5 Infectious and Tropical Diseases Service, María Auxiliadora Hospital, Lima, Peru. Infectious and Tropical Diseases Service María Auxiliadora Hospital Lima Peru; 6 Clinical Pathology Service, Microbiology Area, Hospital María Auxiliadora, Buenos Aires, Argentina, Lima, Peru. Clinical Pathology Service Microbiology Area Hospital María Auxiliadora Lima Peru

To the Editor. Although *Acinetobacter baumannii* is the best-known species of the genus *Acinetobacter*, there are other species that can cause severe infections. Among these, there is a group of phylogenetically similar species indistinguishable by standard procedures from *A. baumannii* that, together with *A. calcoaceticus,* form the *Acinetobacter calcoaceticus-Acinetobacter baumannii* complex (ACB) ^1^. In this letter, we present the first documented case of *A. pittii* / *lactucae* isolation as a cause of hospital infection in Peru.

The sample was isolated from a 23-year-old male patient positive for human immunodeficiency virus (HIV), on antiretroviral treatment and diagnosed with intestinal tuberculosis (date of diagnosis: 07/15/2020), who was admitted in stage 2C (viral load: 40 copies, CD4 count: 231 cells/mL) to the Emergency Department on January 8, 2021, with diffuse abdominal pain, nausea, vomiting and constipation for 5 days, being diagnosed with an intestinal obstruction. On clinical examination, the patient was in poor general condition, hemodynamically unstable, with rebound tenderness and serological markers of sepsis. The CT scan showed free liquid in the cavity and pneumoperitoneum. On 09/01/2021 he underwent surgery for perforation of the cecum, ascending colon and terminal ileum; right hemicolectomy and ileostomy were performed. On postoperative day 13, the ileostomy wound was erythematous, painful on palpation and produced whitish fluid. He received empirical treatment with meropenem and vancomycin, which was then modified to gentamicin (dose of 7 mg/kg every 24 hours for 7 days) and metronidazole based on an antibiogram. The patient was discharged after 33 days, with intestinal transit restitution (Supplementary Table 1). 

The isolate was initially identified as ACB by automated methods (VITEK-2, BioMérieux, Marcy l’Etoile, France), confirmed by MALDI-TOF (MALDI Biotyper®, Bruker Daltonics GmbH & Co. KG, Bremen, Germany), and as a probable *A. pittii* or *A. lactucae* (Table 1 and Supplementary Table 2) by the MBT Compass Library DB-6903 (V.6) (May 2016) and the MBT Compass Library V11.0.0.0 (July 2021). Amplification (Supplementary Table 3) and sequencing of 16S rRNA matched and showed 100.0% similarity to *A. pittii* and 99.5% similarity to *A. lactucae* (Table 1). Due to the high similarity to both species, the isolate was classified as *A. pittii / lactucae*.

**Table 1:** Identification of clinical isolation.

Microorganism	MALDI-TOF		16S rRNA	
	MBT DB-6903 (V6)	MBT V11.0.0.0	%	Identity ^a^
*A. pittii*	2.02	2.05	100.0	1449/1449
*A. lactucae*	NA ^b^	2.18	99.5	1442/1449

a Number of matching bases / total number of bases compared. In both cases the best comparisons according to blastn are indicated.

b Not available. *A. lactucae* is not present in the MBT Compass Library DB-6903 (V6) database.

The isolate showed high levels of resistance and was classified as multidrug resistant and potentially extremely resistant (XDR), being sensitive only to amikacin and colistin, as well as intermediate to gentamicin and tigecycline (Supplementary Table 4). Molecular analysis showed the presence of *bla*_PER_, *bla*_OXA-23G_, *bla*_OXA-24G_, *bla*_NDM_ and *bla*_VIM_ genes (Supplementary Table 3).

The current nomenclature of *A. pittii* was established in 2013, although this species had previously been described as genospecies 3 ^2^. *A. lactucae* was identified in 2016, as a microorganism phylogenetically very close to *A. pittii*^3^. Often, infections by these species are underdiagnosed and mistaken for *A. baumannii* or reported as ACB. The MALDI-TOF technique allows simple and rapid classification of the species of microorganisms, including those that are part of the ACB complex ^2^. This technique has several advantages, such as its good performance, ease of use, the need for a minimum amount of colony and quickness in obtaining results. Among the disadvantages are the high cost of the equipment and the difficulty in identifying closely related microorganisms or those not present in the database; therefore, it is important to specify the version of the database used in MALDI-TOF studies. Thus, while *A. lactucae* was not registered in the MBT Compass Library DB-6903 (V.6) database of May 2016, it already appears in MBT Compass Library V11.0.0.0.0 of July 2021.

Molecular analysis revealed the presence of multiple carbapenemase-encoding genes and an extended-spectrum β-lactamase (BLEE) *bla*_PER_ type. Baraka *et al*. analyzed the genomes of different members of the genus *Acinetobacter* (excluding *A. baumannii*), and found that *A. pittii* harbored up to 28 distinct genes encoding different β-lactamases ^4^. They also showed that those detected in our study had been previously identified in this species ^4^. The presence of up to five β-lactamases (four of them were carbapenemases) shows the potential of these species to acquire multiple resistance mechanisms. Notably, this would be the first confirmed case in Peru of multidrug-resistant *A. pittii* / *A. lactucae* carrying *bla*_PER_, *bla*_OXA-23G_, *bla*_OXA-24G_, *bla*_NDM_, and *bla*_VIM_.

The main limitation of this study is the inability to define whether the isolate was *A. pittii* or *A. lactucae*, or even an unidentified species phylogenetically close to them. Nevertheless, we show the presence of *Acinetobacter* species other than *A. baumannii* in hospital settings. 

In conclusion, we describe presence of multidrug-resistant *A. pittii* / *A. lactucae* in surgical wounds for the first time in Peru. The lack of data on these species in the country is possibly due more to the difficulty of precise identification than to their rarity as infectious agents. Future studies designed to determine their real prevalence rates will allow us to understand the relevance of these other species of the ACB group in Peru and will contribute to a more efficient management of patients.

## References

[1] Vijayakumar S, Biswas I, Veeraraghavan B (2019). Accurate identification of clinically important Acinetobacter spp : an update. Future Sci OA.

[2] Nemec A, Krizova L, Maixnerova M, van der Reijden TJ, Deschaght P, Passet V (2011). Genotypic and phenotypic characterization of the Acinetobacter calcoaceticus-Acinetobacter baumannii complex with the proposal of Acinetobacter pittii sp nov. (formerly Acinetobacter genomic species 3) and Acinetobacter nosocomialis sp. nov. (formerly Acinetobacter genomic species 13TU). Res Microbiol.

[3] Rooney AP, Dunlap CA, Flor-Weiler LB (2016). Acinetobacter lactucae sp nov., isolated from iceberg lettuce (Asteraceae: Lactuca sativa). Int J Syst Evol Microbiol.

[4] Baraka A, Traglia GM, Montaña S, Tolmasky ME, Ramirez MS (2020). An Acinetobacter non-baumannii population study antimicrobial resistance genes (ARGs). Antibiotics.

