# Supplementary material for: First description of an infection by Acinetobacter pitti / lactucae subcomplex in Peru
Source: Rev Peru Med Exp Salud Publica. 2023 Sep 25;40(3):377–8. doi: 10.17843/rpmesp.2023.403.12721 (PMC10953635; doi:10.17843/rpmesp.2023.403.12721)
Supplement: Supplementary material. — Available in the electronic version of the RPMESP. [file rpmesp-40-03-12721-s001.docx]

**Primera descripción de infección por el subcomplejo *Acinetobacter pittii* / *lactucae* en Perú.**

**First description of an infection related to *Acinetobacter pittii* / *lactucae* subcomplex in Peru.**

Carla Andrea Alonso ^1a^ (ORCID: 0000-0002-9497-8573), Jorge Choque-Matos ^2b^ (ORCID: 0000-0001-5273-2009), Fernando Guibert ^3c^ (ORCID: 0000-0001-5282-0148), Beatriz Rojo-Bezares^4d^ (ORCID**: 0000-0003-2742-0980),** María López ^4d^ (ORCID: 0000-0002-3834-4891), Rocio Egoávil-Espejo ^3e^ (ORCID: 0000-0003-2141-9129), Patricia Gonzales (ORCID: 0000-0002-3863-7870) ^5f^, Carmen Valera-Krumdieck ^6g^ (ORCID: 0000-0002-6209-9507, María J. Pons ^3h^ (ORCID: 0000-0001-8384-2315), Yolanda Saénz ^4d^ (ORCID: 0000-0002-2457-4258), Joaquim Ruiz ^3h^ (ORCID: 0000-0002-4431-2036)^2^*

^1^ Departamento de Diagnóstico Biomédico, Laboratorio de Microbiología, Hospital San Pedro, Logroño, España; ^2^ Grupo de Medicina Regenerativa, Universidad Científica del Sur, Lima, Perú; ^3^ Grupo de Investigación en Dinámicas y Epidemiología de la Resistencia a Antimicrobianos - “One Health”, Universidad Científica del Sur, Lima, Perú; ^4^ Área de Microbiología Molecular, Centro de Investigación Biomédica de La Rioja, Logroño, España; ^5^ Servicio de Enfermedades Infecciosas y Tropicales, Hospital María Auxiliadora, Lima, Perú; ^6^ Servicio de Microbiología, Hospital María Auxiliadora, Lima, Perú.

**Tabla Suplementaria 1: Datos básicos del paciente.**

|  | Paciente |
| --- | --- |
| Microorganismo | *Acinetobacter pittii* / *lactucae* |
| Muestra | Secreción de herida |
| Edad | 23 |
| Sexo | Varón |
| Tiempo de hospitalización | 33 días |
| Alta hospitalaria con vida | Si |
| COVID-19 | Negativo |
| Comorbilidades | VIH+ (estadio III-C) |
|  | TBC multisistémica |
|  | Enfermedad de Crohn ^a^ |

TBC; Tuberculosis;

^a^ Diagnóstico posterior al alta.

**Tabla Suplementaria 2: Criterios de interpretación de los resultados obtenidos por MALDI-TOF**

|  | **Puntaje MALDI-TOF** | | | |
| --- | --- | --- | --- | --- |
|  | **≤ 1.699** | **1.700 - 1.999** | **2.000 - 2.299** | **≥ 2.3000** |
| **Género** | No fiable | Probable | Seguro | Seguro |
| **Especie** | No fiable | No fiable | Probable | Altamente Probable |

**Tabla Suplementaria 3: Cebadores utilizados en el estudio**

|  | Cebadores | |  |  |  |
| --- | --- | --- | --- | --- | --- |
| Gen | Directo (5’-3’) | Reverso (5’-3’) | pb | Tª | Ref |
| *bla*_CTX-M_ | CGATGTGCAGTACCAGTAA | TTAGTGACCAGAATCAGCGG | 585 | 60 | ^(1)^ |
| *bla*_GES_ | CTGGCAGGGATCGCTCACTC | TTCCGATCAGCCACCTCTCA | 600 | 56 | ^(2)^ |
| *bla*_PER_ | AGTGTGGGGGCCTGACGAT | GCAACCTGCGCAATRATAGCTT | 725 | 57 | ^(2)^ |
| *bla*_IMI_ | CTACGCTTTAGACACTGGC | AGGTTTCCTTTTCACGCTCA | 482 | 57 | ^(3)^ |
| *bla*_IMP_ | ACAYGGYTTRGTDGTKCTTG | GGTTTAAYAAARCAACCACC | 387 | 57 | ^(2)^ |
| *bla*_KPC_ | GTATCGCCGTCTAGTTCTGC | GGTCGTGTTTCCCTTTAGCC | 636 | 55 | ^(4)^ |
| *bla*_OXA-23G_ | CCCCGAGTCAGATTGTTCAAGG | TACGTCGCGCAAGTTCCTGA | 330 | 57 | ^(2)^ |
| *bla*_OXA-24G_ | GCAGAAAGAAGTAAARCGGGT | CCAACCWGTCAACCAACCTA | 271 | 57 | ^(2)^ |
| *bla*_OXA-48G_ | ATGCGTGTATTAGCCTTATCG | CATCCTTAACCACGCCCAAATC | 265 | 57 | ^(2)^ |
| *bla*_OXA-58G_ | GGGGCTTGTGCTGAGCATAGT | CCACTTGCCCATCTGCCTTT | 668 | 57 | ^(2)^ |
| *bla*_NDM_ | ACTTGGCCTTGCTGTCCTT | CATTAGCCGCTGCATTGAT | 603 | 55 | ^(2)^ |
| *bla*_VIM_ | TGTCCGTGATGGTGATGAGT | ATTCAGCCAGATCGGCATC | 437 | 57 | ^(2)^ |
| 16S rRNA | AGAGTTTGATYMTGGCTCAG^a^ | GGYTACCTTGTTACGACTT^a^ | 1502 | 55 | ^(5)^ |

pb: tamaño del amplicón en pares de bases; Tª: temperatura de hibridación (en ºC); Ref: referencia.

^a^ Cebadores degenerados: Y= C o T; M = A o C.

**Tabla 4: sensibilidad a antimicrobianos**

|  | *A. pittii* / *lactucae* | |  |
| --- | --- | --- | --- |
| Antimicrobiano | CMI | S / I / R |  |
| AMS | 16 | R |  |
| PTZ | ≥128 | R |  |
| CTX | ≥64 | R |  |
| CAZ | ≥64 | R |  |
| CVA | --- | R (17 mm)* |  |
| FEP | ≥64 | R |  |
| ATM | --- | R (9 mm) |  |
| IMP | ≥16 | R |  |
| MEM | ≥16 | R |  |
| CIP | ≥4 | R |  |
| AK | 16 | S |  |
| GEN | 8 | I |  |
| SXT | ≥16/304 | R |  |
| TGY | 4 | I |  |
| COL | ≤0.5 | S |  |

CMI: concentración mínima inhibitoria; S: sensible; I: intermedio; R: resistente; AMS: ampicilina más sulbactam; PTZ: piperacilina más tazobactam; CTX: cefotaxima; CAZ: ceftazidima; CVA: ceftazidima más avibactam; FEP: cefepime; ATM: aztreonam; IMP: imipenem; MEM: meropenem; CIP: ciprofloxacino; AK: amikacina; GEN: gentamicina; SXT: cotrimoxazol; TGY: tigeciclina; COL: colistina.

En todos los casos excepto colistina y ceftazidima más avibactam, la sensibilidad se determinó mediante (VITEK2, BioMérieux, Marcy l'Etoile, France). La sensibilidad a colistina se determino mediante microdilución en agar, mientras que la de ceftazidima más avibactam se determinó mediante difusión en agar. En todos los cssos se sigyuieron las directrices del Clinical and Laboratory Standard Institute.^(6)^

**Referencias Suplementarias**

1. Palma N, Pons MJ, Gomes C, Mateu J, Riveros M, García W, *et al*. Resistance to quinolones, cephalosporins and macrolides in *Escherichia coli* causing bacteraemia in Peruvian children. J Global Antimicrob Resist. 2017;11:28-33. doi: 10.1016/j.jgar.2017.06.011.
2. Bogaerts P, Rezende de Castro R, de Mendonça R, Huang TD, Denis O, Glupczynski Y. Validation of carbapenemase and extended-spectrum β-lactamase multiplex endpoint PCR assays according to ISO 15189. J Antimicrob Chemother. 2013;68:1576-82. doi: 10.1093/jac/dkt065.
3. Mlynarcik P, Roderova M, Kolar M. Primer evaluation for PCR and its application for detection of carbapenemases in *Enterobacteriaceae*. Jundishapur J Microbiol. 2016;9:e29314. doi: 10.5812/jjm.29314.
4. Horna G, Velasquez J, Fernández N, Tamariz J, Ruiz J. Characterisation of the first KPC-2-producing *Klebsiella pneumoniae* ST340 from Peru. J. Glob Antimicrob Resist 2017; 9: 36-40. doi: 10.1016/j.jgar.2016.12.011.
5. Lane DJ. 16S/23S rRNA sequencing. En: Stackebrandt ER, Goodfellow M. Nucleic acid techniques in bacterial systematic, Chichester: John Wiley and Sons; 1991. p115-175.
6. Clinical and Laboratory Standards Institute. Performance Standards for Antimicrobial Susceptibility Testing. Supplement M100 - S31. Wayne: CLSI; 2021.
