# Supplementary material for: First description of an infection by Acinetobacter pitti / lactucae subcomplex in Peru
Source: Rev Peru Med Exp Salud Publica. 2023 Sep 25;40(3):377–8. doi: 10.17843/rpmesp.2023.403.12721 (PMC10953635; doi:10.17843/rpmesp.2023.403.12721)
Supplement: Translation

# Translation: Primera descripción de infección por el subcomplejo *Acinetobacter pittii* / *lactucae* en el Perú

**Authors:** Carla Andrea Alonso, Jorge Choque-Matos, Fernando Guibert, Beatriz Rojo-Bezares, María López, Rocio Egoávil-Espejo, Patricia Gonzales, Carmen Valera-Krumdieck, María J. Pons, Yolanda Saénz, Joaquim Ruiz

**Affiliations:** 1 Departamento de Diagnóstico Biomédico, Laboratorio de Microbiología, Hospital San Pedro, Logroño, España.; 2 Grupo de Medicina Regenerativa, Universidad Científica del Sur, Lima, Perú.; 3 Grupo de Investigación en Dinámicas y Epidemiología de la Resistencia a Antimicrobianos - «One Health», Universidad Científica del Sur, Lima, Perú.; 4 Área de Microbiología Molecular, Centro de Investigación Biomédica de La Rioja, Logroño, España.; 5 Servicio de Enfermedades Infecciosas y Tropicales, Hospital María Auxiliadora, Lima, Perú.; 6 Servicio de Patología Clínica, área de Microbiología, Hospital María Auxiliadora, Lima, Perú.

Sr. Editor. Si bien el *Acinetobacter baumannii* es la especie más conocida del género *Acinetobacter*, existen otras especies que pueden causar infecciones severas. Entre estas, destaca un grupo de especies próximas filogenéticamente e indistinguibles por procedimientos estándar de *A. baumannii* que junto a *A. calcoaceticus* conforman el complejo *Acinetobacter calcoaceticus-Acinetobacter baumannii* (ACB) ^1^. En esta carta, presentamos el primer caso documentado de aislamiento de *A. pittii / lactucae* como causa de infección hospitalaria en Perú.

El aislamiento se realizó de un paciente varón de 23 años con virus de la inmunodeficiencia humana (VIH) positivo, en tratamiento antirretroviral y diagnóstico de tuberculosis intestinal (fecha de diagnóstico: 15/07/2020), que ingresó en estadiaje 2C (carga viral: 40 copias, conteo de CD4: 231 cel/mL) al Servicio de Emergencias el 8 de enero de 2021, presentando dolor abdominal difuso, náuseas, vómitos y constipación por 5 días, siendo valorado como obstrucción intestinal. Al examen clínico, el paciente se encontraba en mal estado general, hemodinamicamente inestable, signo de rebote abdominal y marcadores serológicos de sepsis. Se le realizó una tomografía, que evidenció liquido libre en cavidad y neumoperitoneo. El 09/01/2021 fue intervenido por una perforación de ciego, colon ascendente e íleon terminal, por lo que se realizó una hemicolectomía derecha e ileostomía. En el día postoperatorio 13, se observó herida de ileostomía eritematosa, dolorosa a la palpación y secreción blanquecina. Recibió tratamiento empírico con meropenem y vancomicina, luego fue modificado a gentamicina (dosis de 7 mg/Kg cada 24 horas por 7 días) y metronidazol basado en un antibiograma. El paciente fue dado de alta después de 33 días, realizándose una restitución de tránsito intestinal (Tabla Suplementaria 1).

El aislamiento se identificó inicialmente como ACB mediante métodos automatizados (VITEK-2, BioMérieux, Marcy l’Etoile, France), confirmándose por MALDI-TOF (MALDI Biotyper®, Bruker Daltonics GmbH & Co. KG, Bremen, Alemania), y utilizando las bases de datos MBT Compass Library DB-6903 (V.6) (mayo 2016) y MBT Compass Library V11.0.0.0 (julio 2021) como un probable *A. pittii o A. lactucae* (Tabla 1 y Tabla Suplementaria 2). La amplificación (Tabla Suplementaria 3) y secuenciación del 16S rRNA coincidieron y mostraron un 100,0% de similitud con *A. pittii* y 99,5% con *A. lactucae* (Tabla 1). Debido a la alta similitud con ambas especies, el aislamiento fue clasificado como *A. pittii / lactucae*.

**Tabla 1:** Identificación del aislamiento clínico.

Microorganismo	MALDI-TOF		16S rRNA	
	MBT DB-6903 (V6)	MBT V11.0.0.0	%	Identidad ^a^
*A. pittii*	2,02	2,05	100,0	1449/1449
*A. lactucae*	ND ^b^	2,18	99,5	1442/1449

a Número de bases concordantes / número total de bases comparadas. En ambos casos se indican las mejores comparaciones según blastn.

b No disponible. *A. lactucae* no está presente en la base de datos MBT Compass Library DB-6903 (V6).

El aislado mostró elevados niveles de resistencia y se clasificó como multidrogorresistente y potencialmente extremadamente resistente (XDR), siendo sensible únicamente a amikacina y colistina, así como intermedio a gentamicina y tigeciclina (Tabla Suplementaria 4). Los análisis moleculares mostraron la presencia de los genes *bla*_PER_, *bla*_OXA-23G_, *bla*_OXA-24G_, *bla*_NDM_ y *bla*_VIM_ (Tabla Suplementaria 3).

La nomenclatura actual de *A. pittii* se estableció en 2013, aunque previamente se había descrito a esta especie como genoespecie 3 ^2^. *A. lactucae* fue identificado en 2016, como un microorganismo filogenéticamente muy cercano a *A. pittii*^3^. A menudo, las infecciones por estas especies son subdiagnosticadas y se confunden con *A. baumannii* o se reportan como ACB. La técnica de MALDI-TOF permite clasificar de manera sencilla y rápida los microorganismos a nivel de especie, incluyendo aquellas que forman parte del complejo ACB ^2^. Esta técnica presenta innumerables ventajas, como su alto rendimiento, facilidad de uso, necesidad de una mínima cantidad de colonia y rapidez en la obtención de resultados. Entre las desventajas están, el elevado costo del equipamiento o la dificultad que puede suponer la identificación de microorganismos estrechamente relacionados o no presentes en la base de datos, por ello, es importante precisar la versión de la base de datos utilizada en los estudios de MALDI-TOF. Así, mientras en la base de datos MBT Compass Library DB-6903 (V.6) de mayo de 2016, no estaba registrado *A. lactucae*, este ya aparece en MBT Compass Library V11.0.0.0 de julio de 2021.

El análisis molecular reveló la presencia de múltiples genes codificantes de carbapenemasas y adicionalmente de una β-lactamasa de espectro extendido (BLEE) tipo *bla*_PER_. Baraka *et al.*, analizando los genomas de diferentes miembros del género *Acinetobacter* (excluyendo *A. baumannii*), mostraron que *A. pittii* albergaba hasta 28 genes distintos que codificaban diferentes β-lactamasas ^4^. Asimismo, mostraron que las detectadas en el presente estudio ya habían sido identificadas previamente en esta especie ^4^. La presencia de hasta cinco β-lactamasas (cuatro de ellas carbapenemasas) muestra el potencial de estas especies para adquirir múltiples mecanismos de resistencia. Cabe destacar que, este sería el primer caso confirmado en Perú de *A. pittii / A. lactucae* multidrogorresistente y portador de *bla*_PER_, *bla*_OXA-23G_, *bla*_OXA-24G_, *bla*_NDM_ y *bla*_VIM_.

La principal limitación del presente estudio es la incapacidad de poder definir si el aislado era *A. pittii* o *A. lactucae*, o incluso una especie no identificada filogenéticamente próxima a ellas. No obstante, se muestra la presencia de especies de *Acinetobacter* diferentes a *A. baumannii* en entornos hospitalarios.

En resumen, la presencia de A*. pittii / A. lactucae* multidrogorresistente en herida quirúrgica es descrita por primera vez en Perú. La falta de datos sobre estas especies en el país posiblemente se deba más a la dificultad de identificación precisa que a su rareza como agentes infecciosos. El desarrollo de estudios diseñados para determinar su prevalencia real permitirá conocer la relevancia de estas otras especies del grupo ACB en Perú y contribuirá a un manejo más eficiente de los pacientes.
